# Uncovering Microbial Composition of the Tissue Microenvironment in Bladder Cancer using RNA Sequencing Data

**DOI:** 10.7150/jca.93055

**Published:** 2024-03-04

**Authors:** Ruiqian Yao, Bin Ai, Zeyi Wang, Bing Shen, Geng Xue, Dong Yu

**Affiliations:** 1 School of Health Science and Engineering, University of Shanghai for Science and Technology, Shanghai 200093, China.; 2 Department of Medical Genetics, Naval Medical University, Xiang-Yin Road, 800, Shanghai 200433, China.; 3 Department of Precision Medicine, Translational Medicine Research Center, Naval Medical University, Xiang-Yin Road, 800, Shanghai 200433, China.; 4 Shanghai Key Laboratory of Cell Engineering, Shanghai, China.; 5 Department of Urology, Huadong Hospital, Fudan University, Shanghai, China.; 6 Department of Urology, Shanghai General Hospital Affiliated to Nanjing Medical University, Shanghai, 200080, China.; 7 Department of Urology, Shanghai General Hospital, Shanghai Jiaotong University School of Medicine, Shanghai, 200080, China.

**Keywords:** Bladder cancer, Cancer microbiome, Biological factors, Co-occurrence network analysis

## Abstract

**Purpose:** Bladder cancer (BC) is one of the top 10 common tumors in the world. It has been reported that microbiota can colonize tissues and play important roles in tumorigenesis and progression. However, the current understanding of microorganisms in the BC tissue microenvironment remains unclear.

**Methods:** In this study, we integrated the RNA-seq data of 479 BC tissue samples from seven datasets combined with a range of bioinformatics tools to explore the landscape of microbiome in the BC tissue microenvironment.

**Results:** The pan-microbiome was estimated to surpass 1,400 genera. A total of seven core microbiota (*Bacillus*, *Corynebacterium*, *Cutibacterium*, *Escherichia*, *Halomonas*, *Pasteurella*, and *Streptomyces*) were identified. Among them, ***Bacillus*** was widely distributed in all datasets with a high relative abundance (10.11% of all samples on average). Moreover, some biological factors, including tissue source and tumor grade, were found significant effects on the microbial composition of the bladder tissue. ***Pseudomonas***, ***Porphyrobacter***, and ***Acinetobacter*** were enriched in tumor tissues, while ***Mycolicibacterium*** and ***Streptomyces*** were enriched in patients who showed durable response to BCG therapy. In addition, we established microbial co-occurrence networks and found that the BCG therapy may attenuate the microbiological interactions.

**Conclusions:** This study clearly provided a microbial landscape of the BC tissue microenvironment, which was important for exploring the interactions between microorganisms and BC tissues. The identified specific taxa might be potential biomarkers for BC.

## Introduction

Bladder cancer (BC) is the 10th most common cancer among 36 cancers in 185 countries 1. Although the prognosis of BC patients has significantly improved due to the development of diagnostic techniques and treatment strategies, the probability of recurrence and metastasis is still high. Many risk factors may increase the occurrence of BC, including smoking, host genetics, exposure to occupational chemicals, contaminated drinking water, and infectious schistosomiasis 2. Apart from environmental and genetic risk factors, researchers have become increasingly aware that microorganisms inhabiting the human body play an important role in the maintenance of health and the development of diseases. The role of specific microorganisms in the pathogenesis of cancer has been extensively studied. For example, *Fusobacterium nucleatum* inhibited the killing of various tumors by natural killer (NK) cells 3. The well-known *Helicobacter pylori* induced the degradation of the p53 in gastric epithelial cells, leading to gastric cancer 4.

Several studies have discovered that microorganisms might play a potential role in BC tumorigenesis and therapies. For instance, *Acinetobacter* may be related to BC and has been a potential microbial marker of BC 5. The bacillus Calmette-Guérin (BCG) vaccine, developed from an isolated *Mycobacterium bovis* strain, has been the gold standard for the treatment of BC 6. Intravesical injection of *Lactobacillus rhamnosus strain GG* has been found effective in inducing tumor regression 7. Major studies exploring the microbial composition of BC focused on surrogate materials such as stool or urine, rather than directly from the tumor and surrounding tissue, leading to a weak understanding of microorganisms in the BC tissue microenvironment. Liu has verified the occurrence of bladder microbiota dysbiosis in BC patients by analyzing tissue samples from bladder mucosa 8. However, a comprehensive and systematic knowledge of the microbiome in the BC tissue environment is still lacking. Therefore, an emerging focus of BC research is now to understand how the tumor and surrounding microbiome can influence BC development.

Various omics technologies such as transcriptomics, proteomics, metabolomics, and metagenomics, and their combinations provided new insights into the understanding of the human microbiome and its role in cancer development 9. Among these techniques, our study focused on the whole transcriptome data which was demonstrated to be sensitive in bacterial genus detection 10. Seven datasets derived from the NCBI database were integrated and applied to mine the microbial information hidden in the sequencing reads by a series of bioinformatic pipelines. Several biological factors were further explored to detect their influences on the microbial composition of BC tissues. Finally, we constructed co-occurrence networks to unravel the microbial interactions within and among different groups. These results would not only provide important implications for the subsequent tumor microbiome-related studies but also identify valuable biomarkers for BC.

## Methods

### Data collection

We retrieved a database with the keywords “bladder cancer” and “expression profiling by high throughput sequencing” in the NCBI database. Seven datasets (PRJNA255416, PRJNA534108, PRJNA562495, PRJNA735225, PRJNA688091, PRJNA552055, and PRJNA186504) were screened out for further analysis. The raw fastq files were downloaded and processed for quality control using the software FastQC (version 0.11.9) and Trim Galore (version 0.6.7).

### Identification and quantification of the microbiome

The ultrafast Karen2 algorithm 11 was used to identify the microorganisms. The microbial reference database contains 83,212 genomes, which include almost all known fungal, bacterial, archaeal, and viral genomes. The microbial taxa with less than three reads were regarded as false positives and then discarded. Due to the sensitivity of detection techniques at the genus level and the experience of previous studies 10, 12, the following analyses were aggregated to taxa at the genus level.

### Microbial profile analysis

Core taxa were identified using the microbiome R-package (version 1.16.0) with fixed thresholds: the positivity detection rate was set as 0.1%, and the prevalence was set as 20% 13. Visualization of shared taxa was performed with the UpSet R-package (version 1.4.0). Stochastic cumulative biocurves were generated by the vegan R-package (version 2.6-2) and fitted equations were calculated to estimate the pan-microbiome.

### Diversity metrics and differential abundance analyses

Alpha diversity (Shannon index) and beta divergence were calculated using vegan R-package (version 2.6-2). Differential analysis was determined in diversity using the Wilcoxon signed rank test. PERMANOVA was used to quantify multivariate community-level differences in microbial composition among groups. *P*-value < 0.05 was considered significant at the group level. Statistically significant differences in the relative abundance of taxa were performed using linear discriminant analysis (LDA) effect size (LEfSe, http://huttenhower.sph.harvard.edu/galaxy/). Only taxa with LDA greater than 3.5 at a *p*-value < 0.05 were considered significantly enriched.

### Co-occurrence network analysis

Co-occurrence network analysis was conducted using igraph (version 1.3.1) and psych (version 2.2.5) R-packages. The Spearman's correlations at r > 0.4 and *p*-value < 0.05 were used for network construction. The network properties, clustering coefficient, modularity, average path length, average normalized degree, and betweenness centralization, were analyzed using the “igraph” package. Within-module connectivity (Zi), and among-module connectivity (Pi) of the seven networks were calculated and compared 14.

## Results

### Population characteristics

To investigate the microbial composition of BC, seven RNA-seq datasets with 479 samples were screened out and downloaded from the NCBI database. Of the datasets, 87.34% of the samples were tumor tissue samples and the remainder were paracancerous tissue samples **(Table 1)**. Except for some samples with missing information, this study predominantly consisted of male participants. The mean age of the participants was 67.68, which is the age range with a high incidence of BC. Notably, each dataset had its special focus beyond other common factors: tumor grade for PRJNA255416 and PRJNA534108, tissue source for PRJNA552055 and PRJNA186504, cancer subtype for PRJNA562495 and BCG therapy outcomes for PRJNA735225 and PRJNA688091.

### Microbial presence in BC transcriptome data

**Figure 1:**
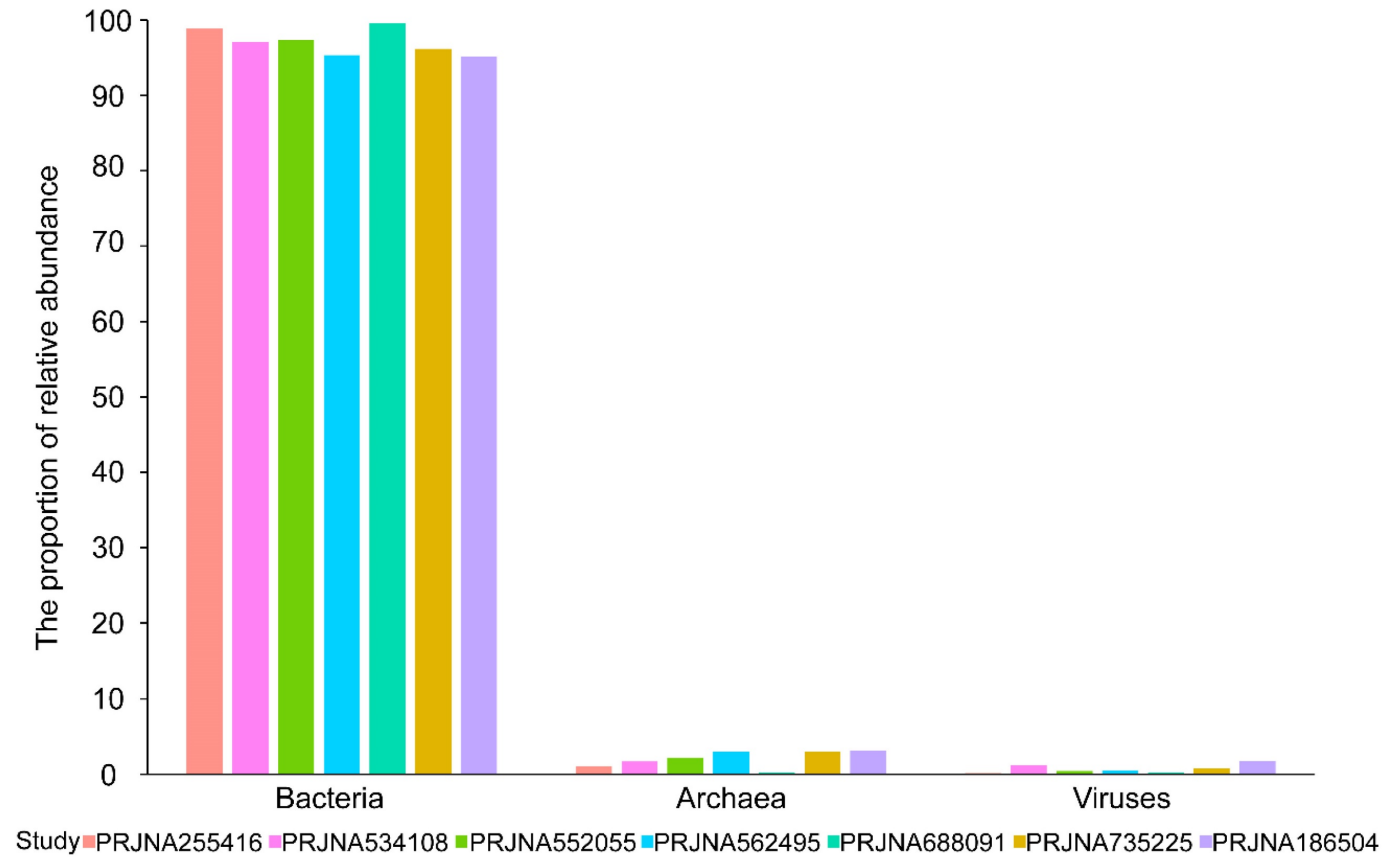
** Microbial abundance histogram of seven datasets.** The proportion (%) of bacteria, viruses, and archaea in identified microbial reads in each dataset.

The microbial reads and corresponding taxa information were identified using the kraken2 algorithm. On average, 0.15% of the 3.99 × 10^7^ sequencing reads per sample were identified as microbes, including bacteria, viruses, and archaea. A total of 5503 microbial taxa at the species level were detected, of which bacteria, viruses, and archaea accounted for 90.62% (4987/5503), 5.23% (288/5503), and 4.14% (228/5503), respectively. The bacterial and viral reads accounted for the majority of microbial reads, while the archaeal reads took a negligible proportion (**Figure 1**). In a word, these results suggested that there indeed are microbial organisms, especially bacteria, located in the microenvironment of BC tissues.

### The characterization of microbial composition in BC tissues

**Figure 2:**
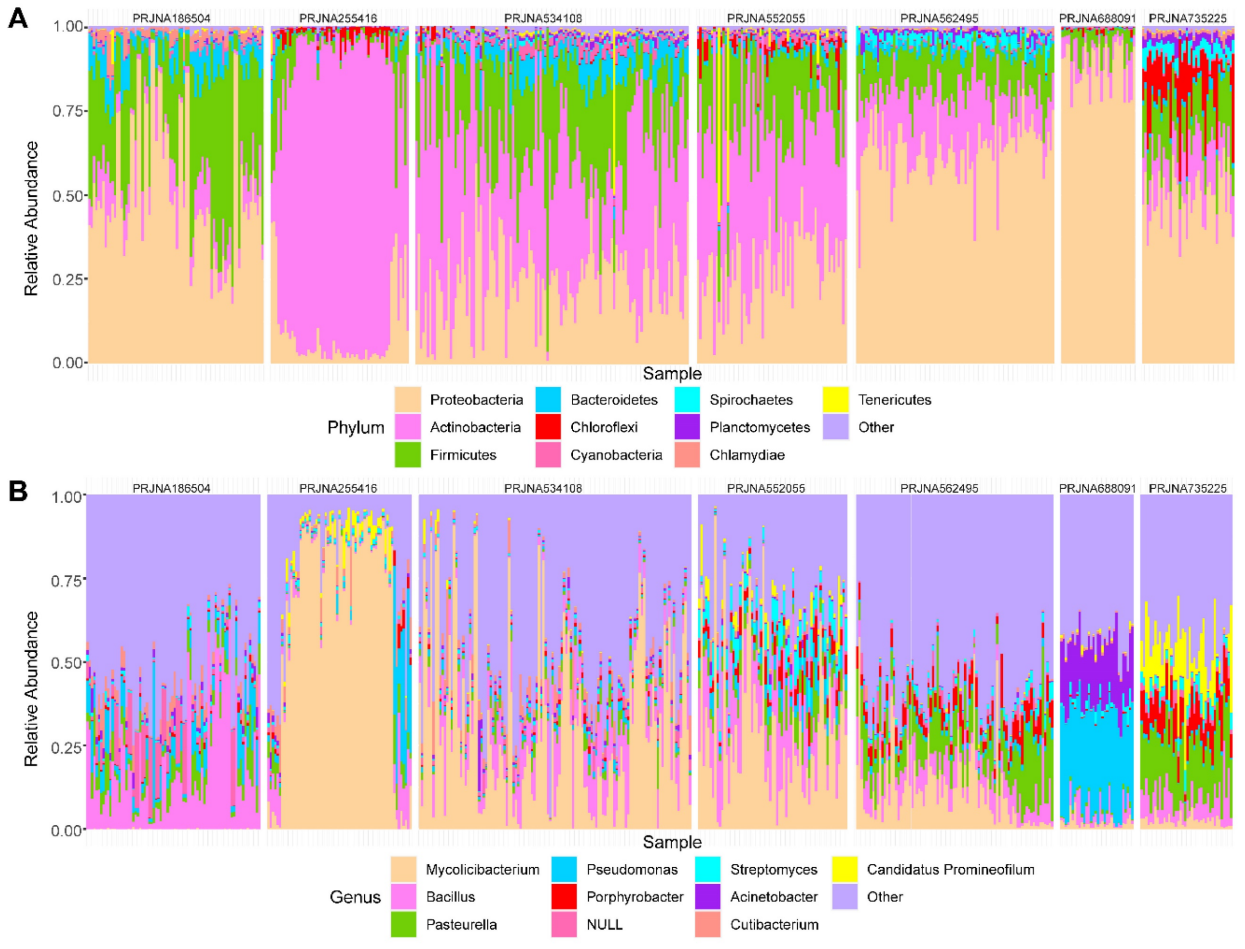
** Different levels of the top ten microbial distributions.** The relative abundances of the top 10 phyla(A) and top 10 genera (B) across the seven datasets.

Next, we further investigated the microbial composition at different levels across the datasets. At the phylum level, a total of 40 phyla were identified, 55% (22/40) of which were shared in seven datasets** (Figure S1A)**. The top 10 phyla in the seven datasets are shown in **Figure 2A**. *Proteobacteria*, *Actinobacteria*, *Firmicutes*, and *Bacteroidea* were found to take the majority of microbial composition in each dataset, which was consistent with 16S sequencing data 15.

Considering the resolution and reliability of the microbial identification method, we assigned all taxa at the genus level. 1313 genera were detected, of which the seven datasets contained 1183, 1306, 899, 510, 832, 898, and 764 genera respectively **(Figure S1B)**. Among the top 10 genera, nine genera were detected in all datasets except *Candidatus Promineofilum* **(Figure 2B)**. *Bacillus* displayed a higher relative abundance in most datasets, which has been reported to be a core genus in BC 5. In addition, *Porphyrobacter* accounted for a higher proportion in PRJNA562495 and PRJNA735225 treated with BCG, which was also consistent with the high throughput 16S rRNA amplicon sequencing data 16.

**Figure 3:**
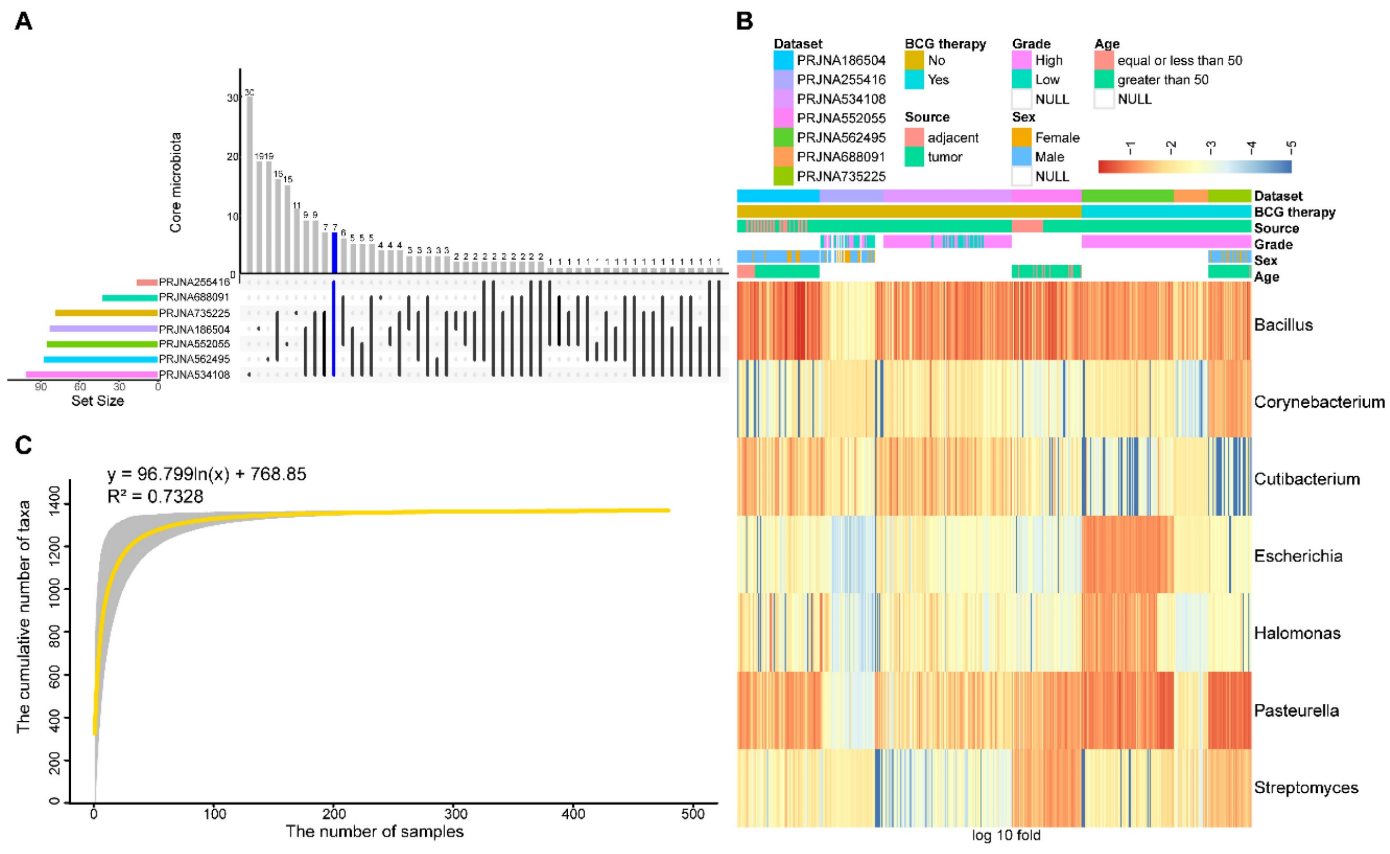
** Core and pan microbiome.** (A) Frequency of shared core microbiota across 7 datasets. 7 taxa (blue highlight) were found to be shared across all 7 datasets. (B) Heatmap of the relative abundance distribution of the seven shared core microbiota in the datasets. (C) Statistic estimation of the size of pan-microbiome. The fitting formula and R2 value are labeled at the top.

To identify differences and commonalities among the datasets, core taxa were characterized. 17, 112, 96, 86, 46, 93, and 91 genera were identified as core microbiota in each dataset, respectively. Among them, seven genera, including *Bacillus*, *Corynebacterium*, *Cutibacterium*, *Escherichia*, *Halomonas*, *Pasteurella*, and *Streptomyces,* were shared among all datasets** (Figure 3A)**. The relative abundance of seven core genera was presented in Figure 3B, as anticipated, these seven core genera were detected in an average of 96.78% of the samples. Among them, *Bacillus* and *Pasteurella* were highly abundant in most samples, while *Cutibacterium* had a low abundance in the BCG-treated dataset.

To estimate the overall number of microbial taxa colonized at the BC tissue, the concept of “Pan-genome” was referred to. With the inclusion of 479 samples, the pan microbial profile in BC tissue appears not to have been reached, as depicted in the accumulation curve and fitting formula** (Figure 3C)**. The size of the pan microbiome of BC probably surpassed 1400 taxa. Therefore, there were an incredible number of microbial taxa colonized in the BC tissue, which should get more attention to explore their roles in tumorigenesis.

### The biological factors affecting the microbial diversity and composition in BC

Considering different biological factors associated with BC in each dataset, we further explored whether these factors had impacts on the microbial composition. Alpha diversity and beta divergence are two measures used to quantify the diversity of a particular microbial community. The LEfSe algorithm for groups with PERMANOVA test* p*-value < 0.05 to identify the differential genus between groups. Genera with a threshold of LDA > 3.5 were defined as significantly different genera **(Table 2)**. In a previous study, Pederzoli reported that male and female patients of BC have different urinary microbiomes 17. However, in our results, no significances were identified between male and female patients in PRJNA255416, PRJNA735225, and PRJNA186504 (PERMANOVA test, *p*-value = 0.130, 0.305, and 0.239). It suggests that, unlike the tissue microenvironment, more factors may be influencing the genitourinary system leading to BC. In contrast, another four factors were identified to have significant effects on the tissue microbiome of BC.

### Tumor grade

The samples were assigned with tumor grade (high-grade vs. low-grade) information in the datasets PRJNA255416 and PRJNA534108. The results showed that tumor grade did not have a significant effect on the microbial composition of PRJNA255416 (PERMANOVA test, *p*-value = 0.45), including alpha and beta divergence analysis **(Figure S2A)**, while a significant effect was found in PRJNA534108 (PERMANOVA test, *p*-value = 0.002). As for diversity, the low-grade group showed significantly higher microbial richness than the high-grade group, while the opposite was true for heterogeneity **(**Wilcox test, *p*-value < 0.01, **Figure S2B)**. 7 genera were then identified to be significantly different between groups** (Table 2)**. Among them, *Mycolicibacterium* was significantly enriched in the high-grade group, while the other six genera, especially *Pasteurella*, were enriched in the low-grade group** (Figure S3A)**.

### Tissue source

Both PRJNA552055 and PRJNA186504 had tumor and paracancerous tissue samples, and the PERMANOVA test showed that there were significantly different microbial composition between tumor and paracancerous tissue samples in both datasets (PERMANOVA test, for PRJNA552055, *p*-value = 0.002; for PRJNA186504, *p*-value = 0.001). In the diversity analysis, the Shannon index of the tumor tissue samples was significantly higher than that of the paracancerous tissue samples in PRJNA552055 (Wilcox test, *p*-value < 0.001, **Figure S2C**-**D**). *Pseudomonas* was significantly enriched in tumor samples in both datasets** (Table 2, Figure S3B)**. Two species of this genus, *P*. *aeruginosa* and *P*. *putida*, have been reported to be significantly enriched in BC compared to normal tissues, which is consistent with our results 13.

### BCG therapy

Intravesical BCG is widely used in the management of BC 6, but the probability of recurrence remains high. In PRJJNA688091, we explored the difference between three clinical outcomes after BCG therapy. The results showed no significant differences in microbial composition among patients with non-relapse, recurrence, and progression (PERMANOVA test, *p*-value = 0.556, 0.641, 0.548). Interestingly, we found that the progression group had significantly higher heterogeneity than the non-relapse group (Wilcox test, *p*-value < 0.05, **Figure S2E)**. In PRJNA735225, we investigated the difference between whether patients responded to BCG therapy or not. A significant effect on the microbial composition between durable and non-durable responders (PERMANOVA test, *p*-value = 0.005) was found. But there were no significant differences in alpha diversity and beta divergence **(Figure S2F)**. Four genera, including *Erythrobacter*, *Corynebacterium*, *Streptomyces*, and *Mycolicibacterium*, were found to be significantly enriched in the durable responder samples, while *Pasteurella* and *Simkania* were enriched in the non-durable responder samples** (Table 2)**.

### Cancer subtype

The samples of dataset PRJNA562495 contain two BC subtypes, including MPBC and UBC. MPBC is a very rare and aggressive variant of BC 18, and was found to have significant effects on the overall microbial composition (PERMANOVA test, *p*-value = 0.001). We found that the Shannon index in the UBC group was significantly higher than that in the MPBC group, while inter-individual divergence was higher in the MPBC group **(**Wilcox test, *p*-value < 0.001, **Figure S2G)**. 10 genera showed significant difference between the two groups by LEfSe** (Table 2)**. *Pasterurella* was the most enriched genus in the MPBC group, while *Halomonas* was the most enriched in UBC. *Halomonas* has been reported as a biomarker both in perihepatic cholangiocarcinoma (pCCA) and distal cholangiocarcinoma (dCCA) with cholelithiasis (CH) controls 19, suggesting that *Halomonas* may be dysregulated among different cancer subtypes.

### Co-occurrence network analysis

To understand the potential interactions among core genera, we constructed co-occurrence networks of the genera in each dataset based on the clustering patterns using significant correlations (Spearman correlation coefficient r > 0.4, *p*-value < 0.05).

The microbial genera constituting each network dominantly belonged to the phylum* Proteobacteria*, and the network characteristics of each dataset were different **(Figure S4, Table 3)**. It is well acknowledged that a higher clustering coefficient corresponds to more active community and stronger interactions among microorganisms. The PRJNA534108 network showed the highest clustering coefficient (0.88). The PRJNA735225 and PRJNA562495, both treated with BCG, had the lowest clustering coefficient, implying BCG treatment might influence the interaction of microorganisms in BC.

**Figure 4:**
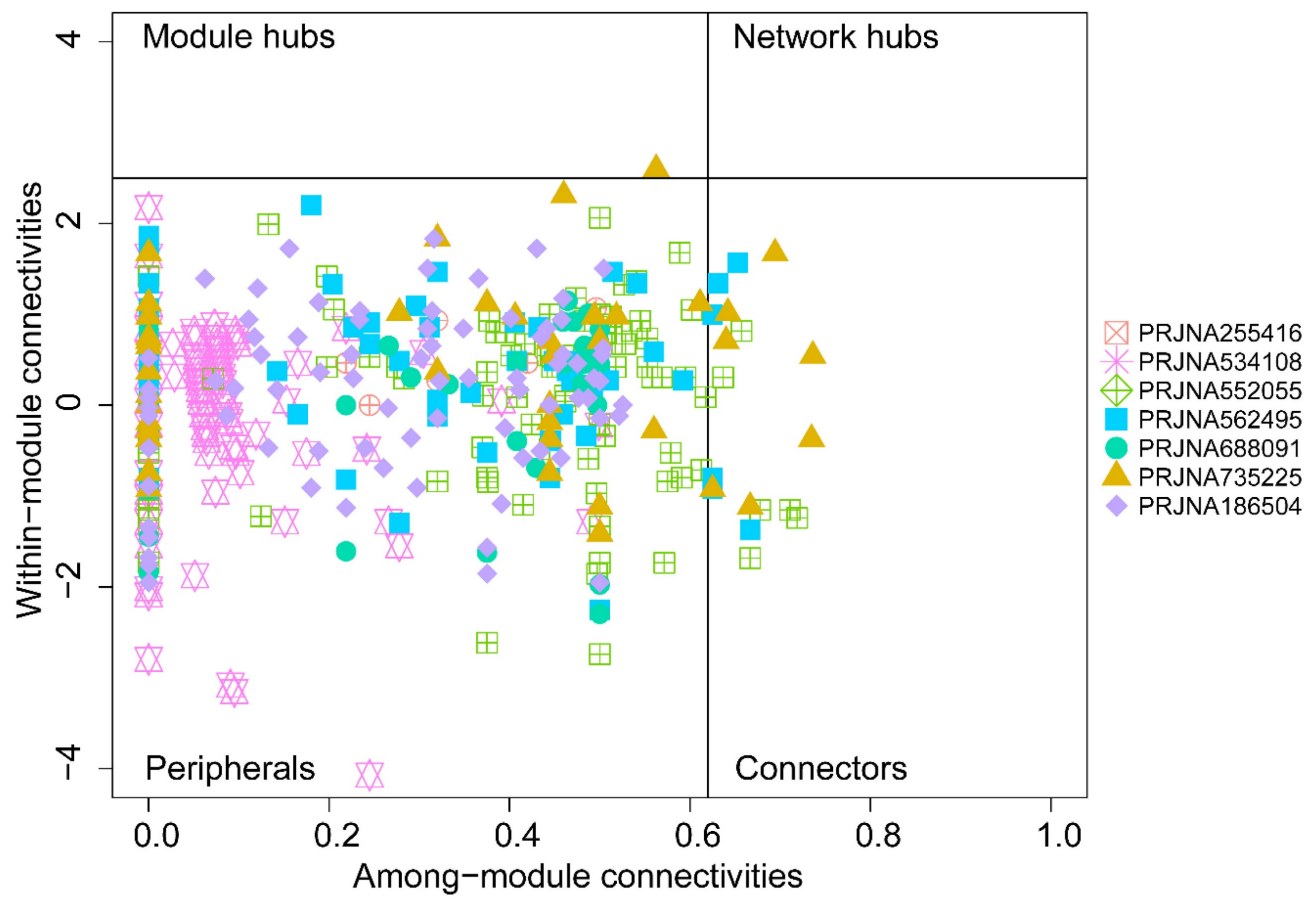
Zi-Pi plot of the individual genera from seven groups.

To assess possible topological roles of taxa in the networks, the nodes were classified into four categories based on Zi and Pi values **(Figure 4)**. Most of the nodes in each network were peripherals. The PRJNA552055, PRJNA562495, and PRJNA735225 networks showed a few connectors. These genera may not simply function as keystone taxa but play important roles in maintaining communication, integrity, and function of tumor microbial communities to the other taxa in the network 20. Three genera, containing *Bartonella*, *Desulfomonile*, and *Pasteurella*, were shared among PRJNA562495 and PRJNA735225. Interestingly, *Bartonella* and *Pasteurella* are both important zoonotic agents, in which *Bartonella* infection can mimic a variety of malignancies 21 and *Pasteurella* has been reported to cause urinary tract infections 22. Moreover, *Chlamydia*, not only acted as the connectors in PRJNA552055 but also acted as the module hubs in PRJNA735225. *Chlamydia* was the cause of common bacterial sexually transmitted infections, including cervicitis and urethritis, and *Chlamydia trachomatis* has been reported as an independent predictor of cervical cancer risk 23.

### Comparison with TCGA cancer microbiome

**Figure 5:**
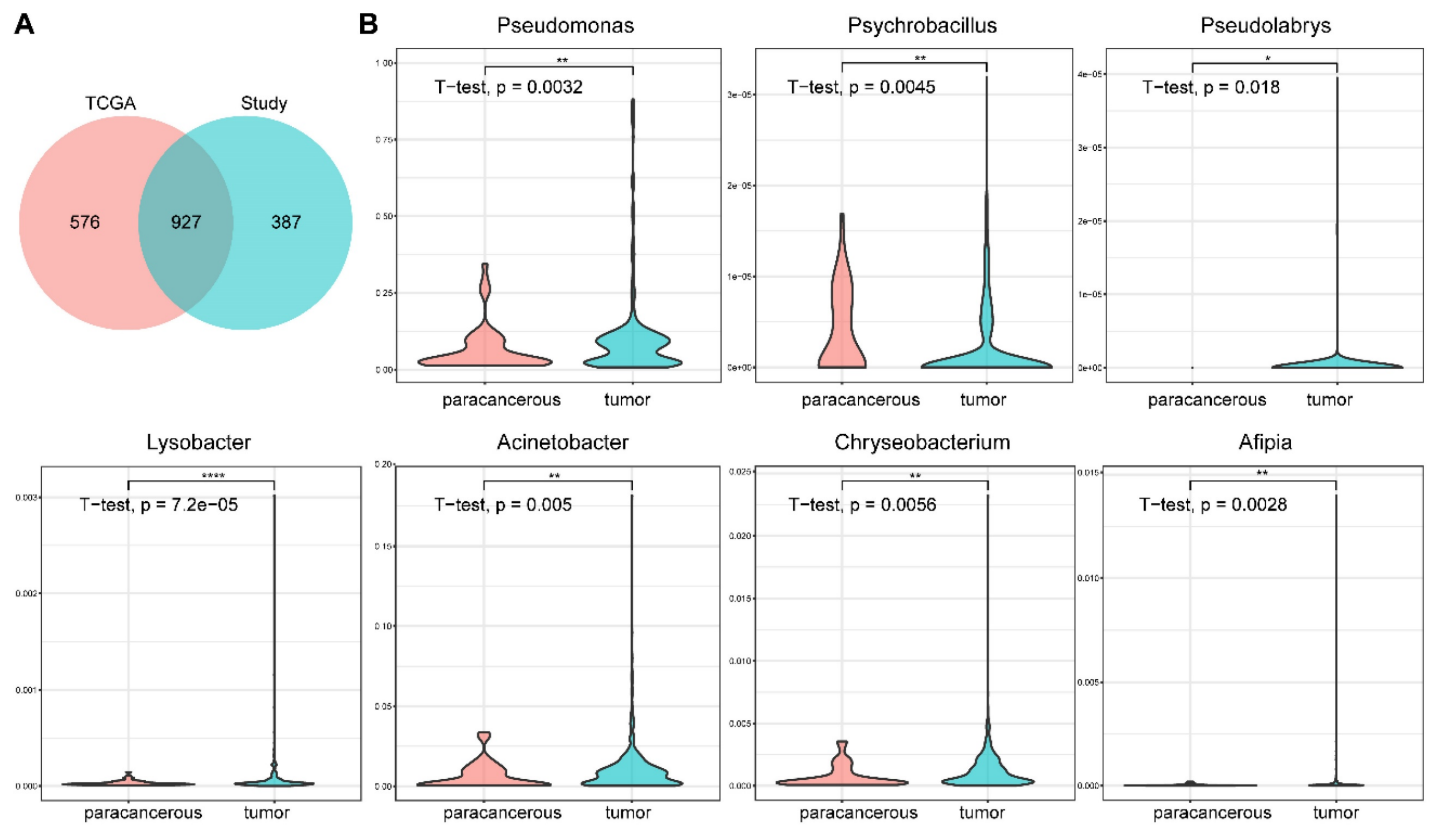
** Comparison between the results of this study and TCGA data.** (A) Overlap of the microbial profiles between this study and TCGA data at the genus level. (B) The relative abundances of seven genera are also significantly different in the TCGA data (tumor vs. paracancerous).

Rob Knight's team analyzed the whole-genome and whole-transcriptome sequencing studies in The Cancer Genome Atlas (TCGA) involving 33 cancer types for microbial reads, including 605 samples from BC 12. Comparing our results with their data revealed some similarities. 61.68% (927/1503) of the genera we found were presented in their results** (Figure 5A)**, and four genera (*Bacillus*, *Escherichia*, *Corynebacterium*, and *Streptomyces*) were also defined as core genera in their dataset** (Figure 5B)**. Seven core genera (*Pseudomonas*, *Psychrobacillus*, *Pseudolabrys*, *Lysobacter*, *Acinetobacter*, *Chryseobacterium*, and *Afipia*), showed significant difference between tumor and paracancerous tissues in our results, were also represented in TCGA (*t-test, p*-value < 0.05, **Figure 5C**). These consistent results indicated the reliability and validity of our analysis.

## Discussion

A growing body of research now suggests that the microbial in tumor and adjacent tissues can inform disease progression and bacterial roles in cancer pathogenesis 13. Most studies have focused on the urine microbiome. However, the composition of the microbiome in the BC tissue microenvironment remains unclear. Based on this, we integrated seven datasets to demonstrate the microbial landscape of BC tissue, including the influencing biological factors and the network of microbial interactions. Novel insights may facilitate the understanding of the role of microbiome in BC tumorigenesis and enable the development of novel therapeutic strategies.

Seven core genera were identified that have been reported to be associated with bladder and other cancers. Among them, *Bacillus* and *Corynebacterium* have been confirmed as the core genera of BC in a 16s RNA sequencing study (direct sequencing) 5. The abundance of *Cutibacterium* was decreased after BCG treatment, and it was found to be associated with BCG effectiveness 24. *Escherichia* has been validated to promote BC through epithelial-mesenchymal transition, stemness, and metabolic reprogramming 25. *Pasteurella* has been reported to cause urinary tract infections 22. A 146 kDa protein toxin produced by* Pasteurella* has been considered carcinogenic due to its high mitogenic activity 26. In addition, two new findings, *Streptomyces* and *Halomonas*, were reported to be associated with cholangiocarcinoma and colorectal cancer respectively 24, 27. These evidences suggested that these core genera might play a potentially important role in BC and other cancers, implying that more attention should be paid to exploring the function and mechanism of these core genera on the development of BC.

Further, we identified a series of significantly differential genera in different biological factor groups. *Pseudomonas* were more enriched in low-grade tumor tissues than in high-grade tumor tissues, which implied that *Pseudomonas* might be a potential biomarker to predict the malignant degree of the tumor. Meanwhile, two species *Pseudomonas aeruginosa* and *Pseudomonas putida*, belong to *Pseudomonas*, have been reported to be significantly enriched in tumor tissues compared with paracancerous tissues, which is consistent with our results 13. Antibiotics are known to have antibacterial effects to a certain extent 28. However, a recent study found that the use of fluoroquinolone antibiotics was significantly associated with high recurrence rates of BC (HR 3.28, 95% CI 1.12-9.60; *p* = 0.03) 29, which may be due to the resistance of *Pseudomonas aeruginosa* to fluoroquinolones 30. It has been reported that *Pseudomonas aeruginosa* is resistant to a variety of antibiotics 31, whereas meropenem-levofloxacin combination therapy has been found to have a better inhibitory effect on *Pseudomonas aeruginosa*, suggesting that this may be a new idea for the treatment of BC 32. Interestingly, not only was *Porphyrobacter* significantly enriched in tumor tissues, but it was also considerably more abundant in the MPBC group compared to the UBC group. It has been reported to be significantly enriched in the higher risk of progression group with BC during treatment 16, which suggested that *Porphyrobacter* might participate in tumor recurrence of BC. *Acinetobacter*, significantly enriched in tumor tissue samples in our results, has been reported to be considered as a potential microbiological marker for BC 5. Meanwhile, *Mycolicibacterium* significantly enriched in the durable response to BCG therapy group, has been reported decreased as the progress of lymph node metastasis in the pancreatic adenocarcinoma 33. This suggests that *Mycolicibacterium* are associated with cancer development and may provide clues for future treatment of BC. In a word, the key genera presented in the microenvironment of BC tissue may provide insight into the molecular mechanisms of BC. Alfano et al. have proposed that it is important to understand the roles of extracellular matrix (ECM) and microbiota in the development and progression of urothelial carcinomas 34. For example, the outer membrane protein A of *Pasteurella multocida* induces changes in the transcriptome of alveolar macrophages, which are associated with the ECM 35. *Pseudomonas aeruginosa* can infiltrate the ECM by secreting alkaline proteases under anaerobic conditions in vitro (e.g., tumor tissue) 34. InvL, an adhesin required for the uropathogenic process of *Acinetobacter baumannii,* has been reported to bind ECM components and mediate adhesion to urinary tract cell lines 36. These studies indicate that investigating the interaction between bacteria and the ECM may reveal new or dysregulated pathways associated with BC.

Moreover, the co-occurrence of microorganisms can be modeled using network analysis to illustrate microbial relationships and responses to variations of operational factors, like predicting the potential effects of chemotherapy in patients with colorectal cancer 20. Our study used co-occurrence network analysis to find that the BCG therapy may attenuate the microbiological interactions. The co-occurrence network can also observe different topological characteristics of the individual microorganisms, which may imply their biological roles and functions. Some zoonotic agents (*Bartonella* and *Pasteurella*) were defined as connecters, strongly correlated with other genera, suggesting that they may be important functional performers in communities. Furthermore, *Chlamydia* displayed a dual network function and has been recognized as an independent predictor of the risk of cervical cancer 23. This necessitates further investigation and consideration.

However, this study has a few limitations. One is the limited sample size. Therefore, the study examined the between-group variation in a single dataset and then explored commonalities and differences between different datasets to reduce the impact of individual heterogeneity on the results. Another is that contamination is an issue in this type of study. This study mainly focused on the analysis of core microbiota to minimize the impact of potential contaminants on the study.

## Conclusion

In this study, the landscape of microbial composition of BC tissue through integrating seven public datasets is presented for the first time. Seven core microbiota (*Bacillus*, *Pasteurella*, *Cutibacterium*, *Escherichia*, *Corynebacterium*, *Halomonas*, and *Streptomyces*) were identified to be prevalent and abundant across the seven datasets, which should be paid more attention. Moreover, sample source, tumor grade, tissue source, BCG therapy, and cancer subtype showed significant effects on tumor microbiome. Microbial interactions were found to be weaker in the BC microenvironment with BCG therapy. The significantly differential genera (*Acinetobacter*, *Pseudomonas*, *Pasteurella*, *Porphyrobacter*, *Mycolicibacterium*, and *Streptomyces*) may be involved in tumor progression as potential characteristic genera. These results will provide valuable data support for clinical translational applications, including early tumor screening and diagnosis. Additionally, the significantly differential genera will serve as candidate targets for the experimental validation of the molecular mechanisms of microbial action in BC.

## Supplementary Material

Supplementary figures.

## Figures and Tables

**Table 1:** An overview of meta information of seven BC datasets.

Study	PRJNA255416	PRJNA534108	PRJNA562495	PRJNA735225	PRJNA688091	PRJNA552055	PRJNA186504
	(60)/CAN	(119)/CAN	(87)/ESP	(40)/USA	(32)/KOR	(65)/CHN	(76)/CHN
Case Source	Urothelial bladder cancer (UBC)	Urothelial bladder cancer (UBC)	Micropapillary bladder cancer (MPBC)/23 Urothelial bladder cancer (UBC)/63	Urothelial bladder cancer (UBC)	Urothelial bladder cancer (UBC)	Urothelial bladder cancer (UBC)	Urothelial bladder cancer (UBC)
**Sample Source**							
FFPE	56	119	87	40	/	/	/
Fresh Frozen	4	/	/	/	32	65	76
**Sex**							
Male	28	/	75	31	26	/	66
Female	14	/	12	9	6	/	10
NULL	18	119	/	/	/	65	/
**Age**							
Min~Max,median	**/**	**/**	**/**	46~82,69	24~81,72	43~82,64	25~87,66
**Tumor stage**							
pTa	35	34	/	/	/	/	/
pT1	7	85	87	40	32	/	/
pT2	8	/	/	/	/	/	/
pTx	2	/	/	/	/	/	/
No-staging	8	/	/	/	/	/	/
**Tumor Grade (WHO 2004)**							
High	**17**	**93**	87	40	32	/	/
Low	**19**	**26**	/	/	/	/	/
NULL	24	**/**	/	/	/	65	76
**Tissue Source**							
Tumor	60	119	87	40	32	**36**	**44**
Paracancerous	/	/	/	/	/	**29**	**32**
**BCG therapy**	No	No	Yes	**non_durable 23**	**Recurrence 9**	No	No
				**durable 17**	**Non-relapse 15**		
					**Progression 8**		

“/” means NA, which indicates that the dataset does not possess the characteristic.

**Table 2:** Summary of LEfSe analysis results.

Biological factors	Datasets	Differential genus			
Tumor high grade	PRJNA534108	Mycolicibacterium^33^	↑	Paenibacillus^37^	↓
				Pasteurella^26^	↓
				Brevibacillus^38^	↓
				Rothia^39^	↓
				Pseudomonas^13^	↓
				Porphyrobacter^16^	↓
Tumor tissue source	PRJNA552055	Pasteurella^26^	↑	Mycolicibacterium^33^	↓
		Porphyrobacter^16^	↑		
		Streptomyces^27^	↑		
		**Pseudomonas^13^**	↑		
	PRJNA186504	Psychrobacillus^40^	↑	Chlamydia^23^	↓
		Rhodoplanes^41^	↑	Bartonella^21^	↓
		Pseudolabrys^42^	↑	Rummeliibacillus^43^	↓
		Lysobacter^44^	↑	Micrococcus^45^	↓
		Comamonas^46^	↑	Paenibacillus^37^	↓
		Desulfarculus^47^	↑	Bosea^48^	↓
		Streptomonospora^49^	↑	Oligotropha^50^	↓
		Acinetobacter^5^	↑	Afipia^51^	↓
		Mycoplasma^52^	↑	Erythrobacter^53^	↓
		Delftia^54^	↑	Alicycliphilus^55^	↓
		**Pseudomonas^13^**	↑	Brevibacillus^38^	↓
		Methylorubrum^56^	↑		
		Chryseobacterium^46^	↑		
BCG therapy by the response to durable	PRJNA735225	Erythrobacter^53^	↑	Pasteurella^26^	↓
		Corynebacterium^5^	↑	Simkania^57^	↓
		Streptomyces^27^	↑		
		Mycolicibacterium^33^	↑		
MPBC tissue	PRJNA562495	Pasteurella^26^	↑	Halomonas^19^	↓
		Porphyrobacter^16^	↑	Mycolicibacterium^33^	↓
		Streptomyces^27^	↑	Ralstonia	↓
				Enterobacter^58^	↓
				Bacillus^5^	↓
				Staphylococcus^59^	↓
				Kosakonia^60^	↓

The arrows indicate the up-regulation or down-regulation of this genus in this biological factor and studies correlating the genus with tumor or disease development are cited.

**Table 3:** Key characteristics of co-occurrence networks of seven groups.

Dataset	Clustering coefficient	Random Network clustering coefficient	Modularity	Average normalized degree	Betweenness centralization
PRJNA255416	0.72	0.41	0.25	0.42	0.24
PRJNA534108	0.88	0.46	0.11	0.46	0.07
PRJNA552055	0.69	0.24	0.15	0.24	0.06
PRJNA562495	0.56	0.10	0.42	0.10	0.13
PRJNA688091	0.79	0.50	0.11	0.50	0.06
PRJNA735225	0.24	0.05	0.52	0.05	0.19
PRJNA186504	0.64	0.29	0.28	0.29	0.05
