# Supplementary material for: Uncovering Microbial Composition of the Tissue Microenvironment in Bladder Cancer using RNA Sequencing Data
Source: J Cancer. 2024 Mar 4;15(8):2431–41. doi: 10.7150/jca.93055 (PMC10937280; doi:10.7150/jca.93055)
Supplement: Supplementary file 1 — Supplementary figures. [file jcav15p2431s1.pdf]

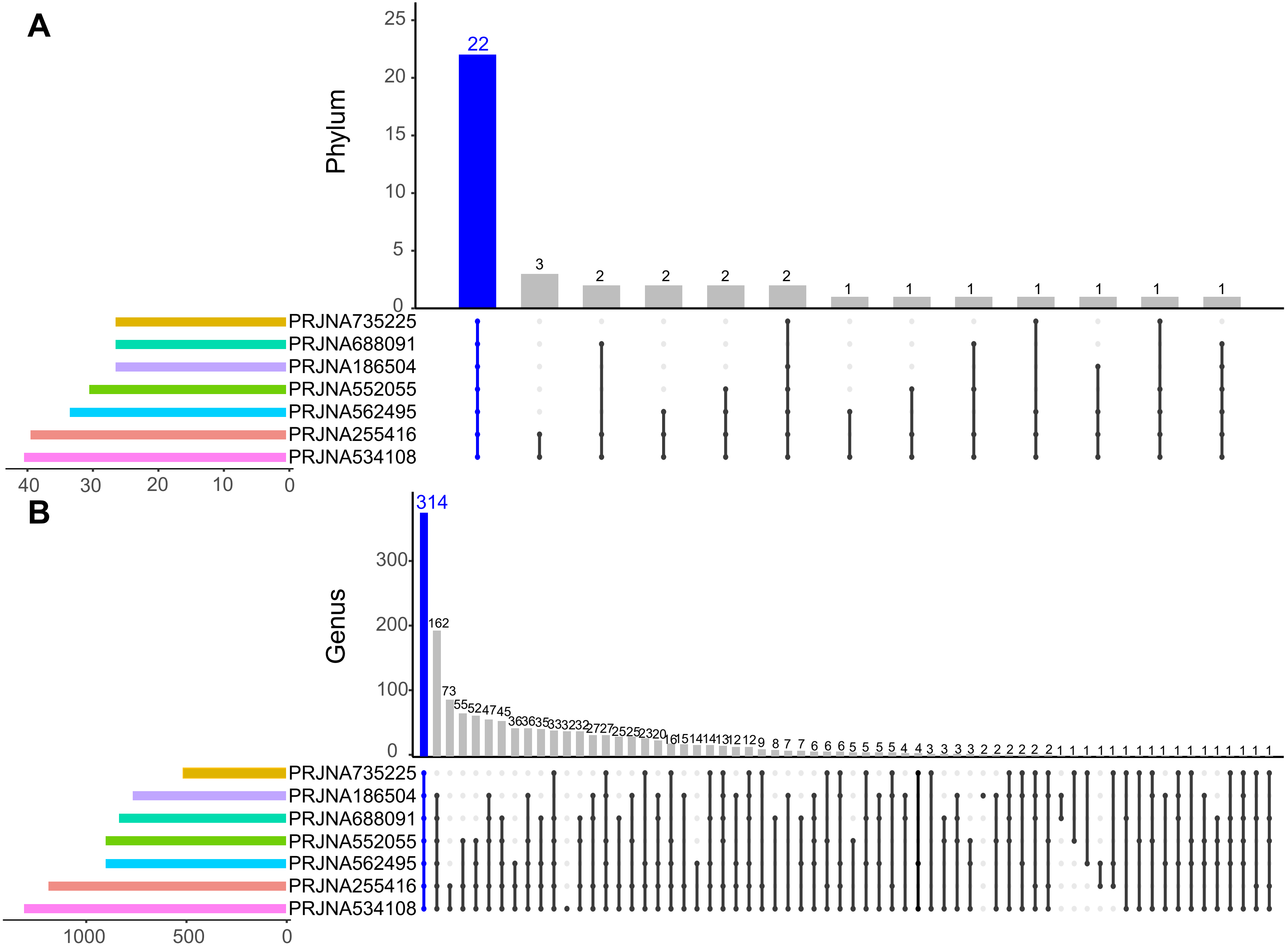

Figure S1: Overlap of the microbial profiles across the seven datasets at the phylum level (A) and the genus level (B).

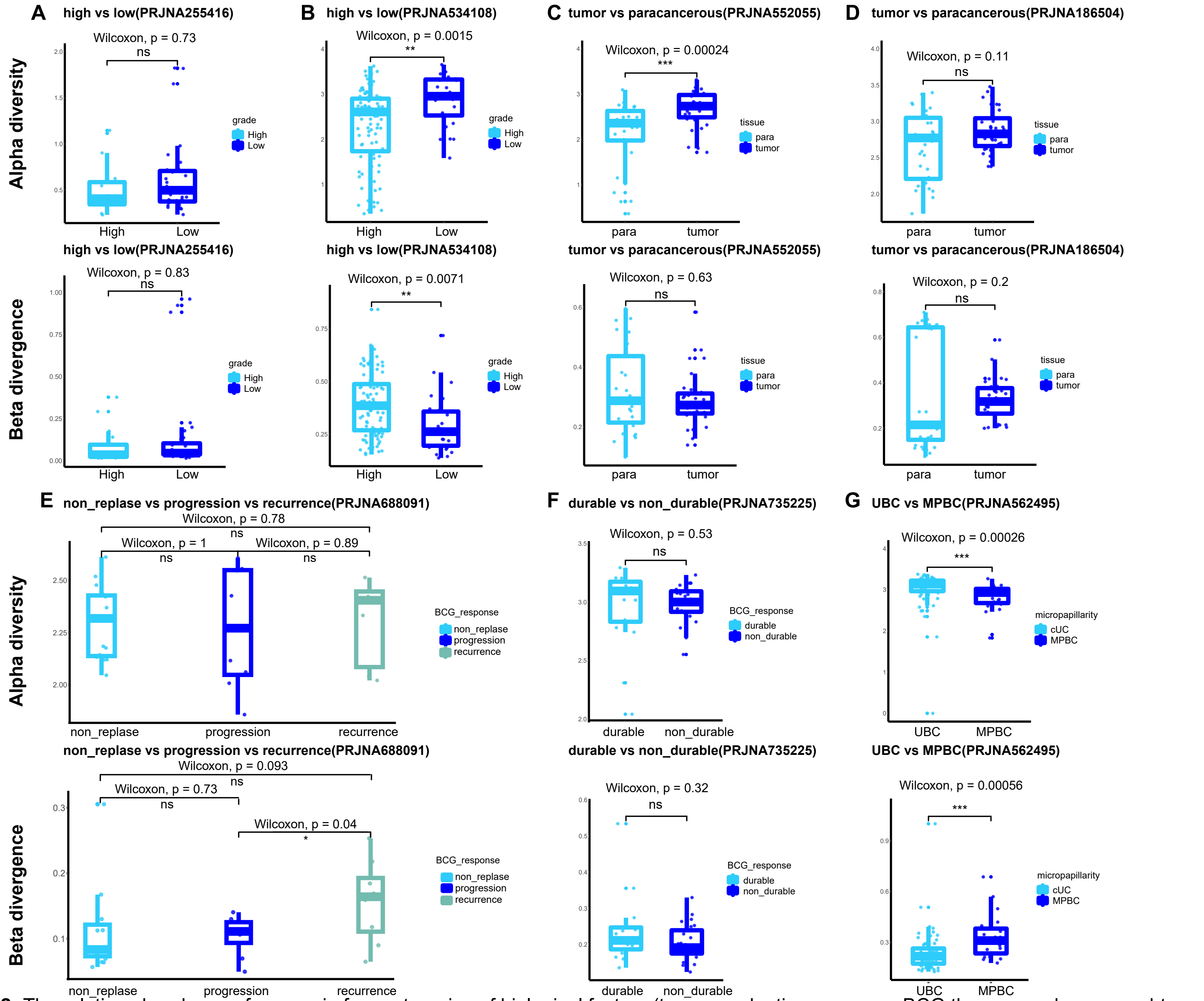

Figure S2: The relative abundance of genera in four categories of biological factors (tumor grade, tissue source, BCG therapy, and cancer subtype was used to calculate the diversity index of Alpha and Beta, and the *p*-value was calculated by Wilcoxon test.

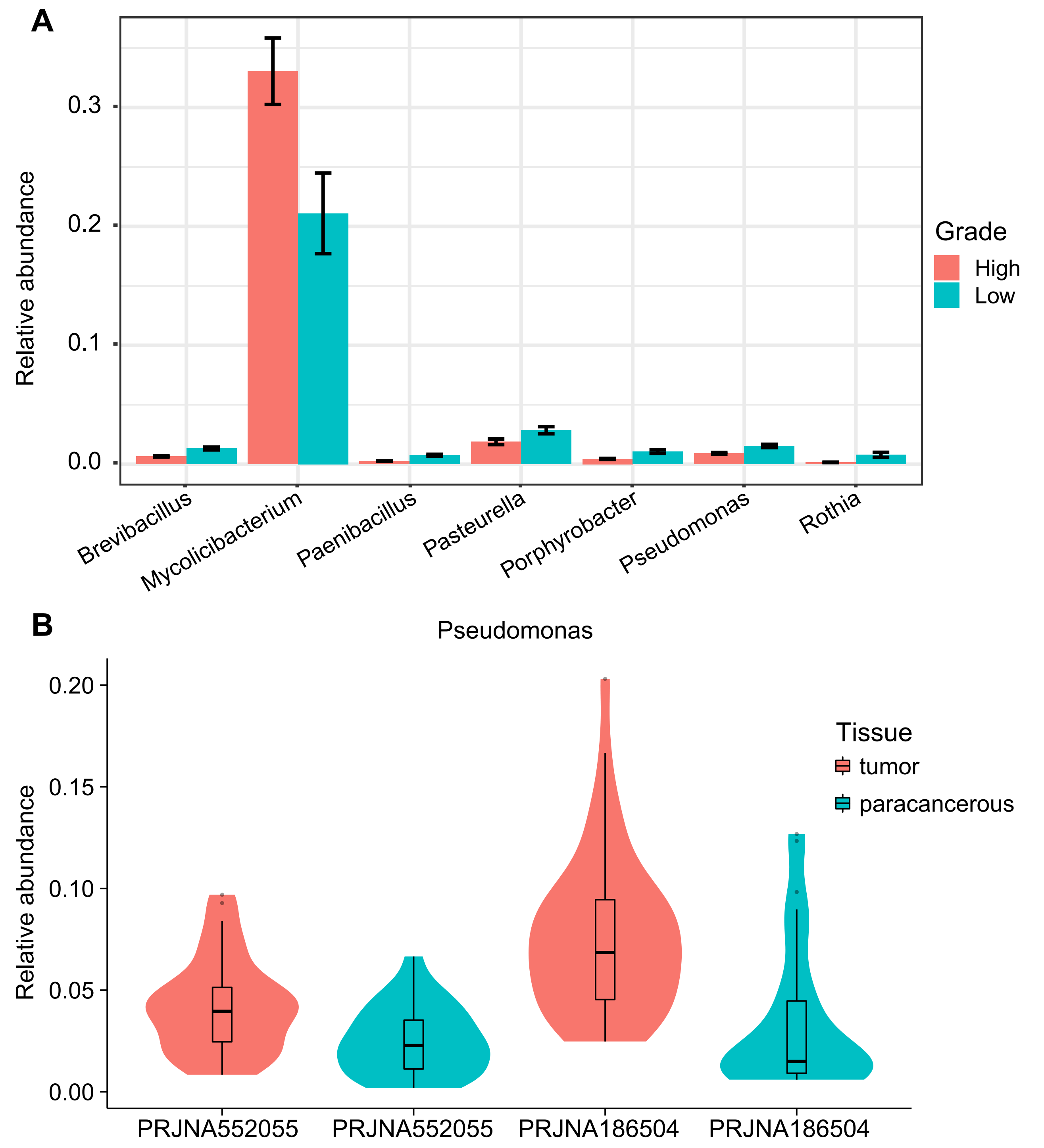

Figure S3: The abundance of some significantly different genera discriminated by LEfse is shown. (A) The bar chart of average relative abundances of significantly different genera in the tumor grade group (High grade vs. Low grade). (B) The relative abundances of differential genus Pseudomonas shared by the two datasets from tissue source(tumor vs. paracancerous).

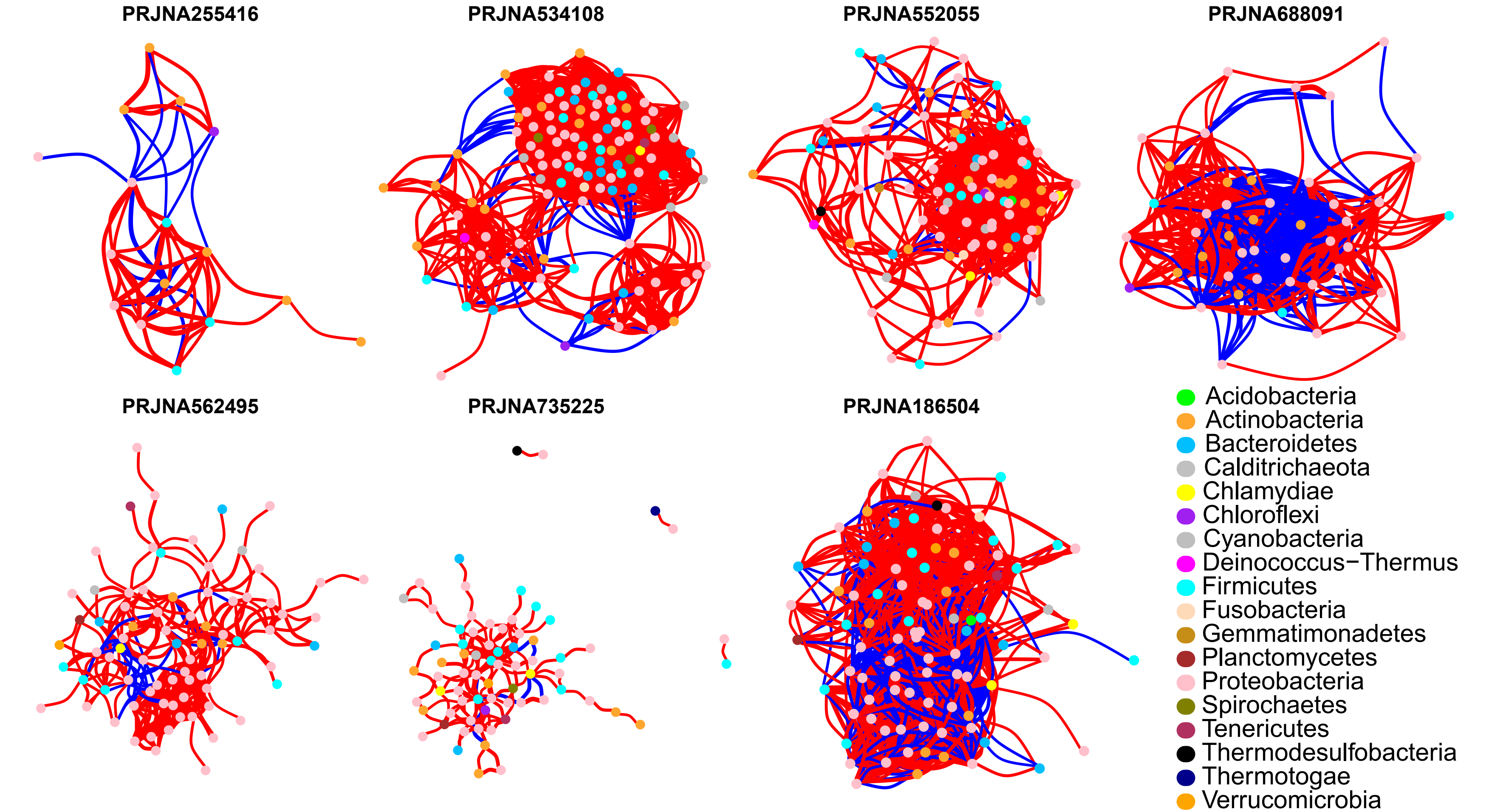

Figure S4: The co-occurrence network of seven groups. The color of nodes indicates genera from the same phylum in each network. Line color indicates positive (red) and negative (blue) correlation coefficient. Spearman's correlation coefficient r > 0.4 and *p*-value < 0.05 were used for network construction.
